# Editorial Department

**Published:** 1857-12

**Authors:** 


### EDITORIAL DEPARTMENT.

The undersigned, Committee of American Medical Association on Influence of Marriages of Consanguinity upon Offspring, solicits aid from every part of the Union in collecting facts upon this interesting subject.

The points of information more especially desired, are:

1st. The degree of consanguinity of parties, and whether by father or mother's side.

(As first, second, third or fourth cousins, or more rarely brother and sister or uncle and niece.)

2d. The approximate date of marriage.

(This is only requisite in order to show whether the fruitful period is past and the case complete as to its results.

.3d. The constitutional diathesis and temperament of *each* parent, their occupation and such habits or circumstances as are, in the opinion of the observer, calculated to influence the normal development of offspring.

(Where the parties are personally known, the color of the hair and eyes and complexion are requested to be noted.)

4th. The number, sex and *condition* of children produced by such marriages.

5th. The number and sex of children who have died, their ages at death and causes, where known.

It is probable that in very many cases all the points of information required above cannot be obtained; it is hoped that those who favor the purposes of the report, will not suffer an inability to procure *all* the facts to deter them from furnishing such as are within their reach. The committee desires to accumulate a mass of statistical truths, whose aggregate will determine beyond question, whether marriages of consanguinity are in the main prejudicial to offspring or not; that he may accomplish his purpose, he begs of his co-laborers to report all instances of such marriage within their circles of observation, whatever their results: whether sterile, or followed by well-developed or imperfect issue.

Contributions are requested to be forwarded as early as practicable, but will be accepted until the first of April next.

(N. B. Names of parties not desired, as all personal allusion will be avoided by which either contributors or their subjects of observation may be designated.)

S. M. Bemiss, M. D.

Louisville, Ky.

We publish the preceding which we received from Dr. Bemiss, hoping that those of the profession who have it in their power will respond promptly to the call. F.

